# Estimating the incidence of norovirus acute gastroenteritis among US and European international travelers to areas of moderate to high risk of traveler’s diarrhea: a prospective cohort study protocol

**DOI:** 10.1186/s12879-018-3461-6

**Published:** 2018-12-03

**Authors:** Lisa Lindsay, Herbert L. DuPont, Christine L. Moe, Martin Alberer, Christoph Hatz, Amy E. Kirby, Henry M. Wu, Thomas Verstraeten, Robert Steffen

**Affiliations:** 1 P95 Pharmacovigilance and Epidemiology Services, Koning Leopold III Laan 1, 3001 Leuven, Belgium; 2 University of Texas McGovern Medical School and School of Public Health, 1200 Pressler Street, Houston, TX 77030 USA; 3 Emory University, Rollins School of Public Health, 1518 Clifton Road NE, Atlanta, GA 30322 USA; 4 Division of Infectious Diseases and Tropical Medicine, University Hospital, Ludwig-Maximilians-University (LMU) Munich, Leopoldstrasse 5, 80802 Munich, Germany; 5 Swiss Tropical and Public Health Institute, Socinstrasse 57, 4056 Basel, Switzerland; 6 University of Basel, Petersplatz 1, 4001 Basel, Switzerland; 7 Emory University, Division of Infectious Diseases, Department of Medicine, 550 Peachtree Street NE MOT 7, Atlanta, GA 30308 USA; 8 University of Zurich; Epidemiology, Biostatistics and Prevention Institute, WHO Collaborating Centre for Travellers’ Health, Hirschengraben 84, 8001 Zurich, Switzerland

**Keywords:** Norovirus, Acute Gastroenteritis, Travel, Traveler’s Diarrhea, Diarrhea, Cohort, Protocol, Epidemiology

## Abstract

**Background:**

Acute gastroenteritis (AGE) is the leading cause of illness among returning travelers seeking medical care. Multiple types of enteric pathogens can cause travel-acquired AGE and, while bacterial pathogens have a predominant role, the importance of viruses, such as norovirus, is increasingly recognized. There is a lack of information on travel-acquired norovirus incidence among symptomatic and asymptomatic individuals irrespective of healthcare-seeking behavior. Our aim is to estimate the incidence of travel-acquired AGE due to norovirus and to characterize the burden of disease among international travelers from the United States and Europe.

**Methods:**

We describe a prospective cohort study implemented in five US and European sites to estimate the role of AGE due to norovirus among adult international travelers. We enrolled individuals aged 18 years and older who are traveling to regions of moderate-high risk of AGE, or via cruise ship with an international port stop, with a trip duration of 3–15 days. The study will generate a wide range of health and travel-related data for pre-, during, and up to 6-months post-travel. We will identify laboratory-confirmed travel-acquired norovirus infections among both symptomatic and asymptomatic individuals from self-collected whole stool samples tested via quantitative RT-PCR. Coinfections will be identified in a subset of travelers with AGE using a multiplex molecular-based assay.

**Discussion:**

This study is unique in design and breadth of data collected. The prospective collection of health and behavioral data, as well as biologic samples from travelers irrespective of symptoms, will provide useful data to better understand the importance of norovirus AGE among international travelers. This study will provide data to estimate the incidence of norovirus infections and AGE and the risk of post-infectious sequelae in the 6-month post-travel period serving as a baseline for future norovirus AGE vaccination studies. This study will contribute valuable information to better understand the role of norovirus in travel-acquired AGE risk and the impact of these infections on a broad set of outcomes.

## Background

Acute gastroenteritis (AGE) remains an extremely common problem among the general population and among international travelers. In international travelers from high- to low-resource settings, poor local sanitation and hygiene result in transmission of enteric pathogens to susceptible travelers [1–4]. Travelers’ diarrhea (TD) (loose/watery stools as dominate symptom while traveling or upon return which may include other symptoms, e.g. abdominal cramps, nausea, vomiting) has been estimated to occur in up to 50% of international travelers during the initial 2 weeks of travel, depending upon study methods, population, and destination(s) [5–9]. In AGE as a broader term, vomiting and other acute abdominal symptoms may be the leading symptoms with or without diarrhea. While there is evidence that TD incidence has declined in many regions, reported incidence rates for travelers to resource-limited destinations still exceed 20% in the initial 2 weeks. TD results in a substantial proportion of incapacitation among patients, but there are limited data in many areas [10, 11]. AGE — usually described as diarrhea — is still the leading diagnosis of ill returning travelers seeking medical care [12–16], as well as illness while abroad among cohorts of travelers from high-resource settings [17, 18].

Pathogens implicated in travel-acquired AGE include bacteria, parasites and viruses [19–21]. Our understanding of their individual contribution reflects endemic circulation within the travel destination, as well as study design and diagnostics such as choice of study population, targeted pathogens, test performance for targeted pathogens, and adequacy of biological sample(s) with respect to optimal detection. Bacterial pathogens, such as Enterotoxigenic *Escherichia coli* (ETEC), Enteroaggregative *E. coli* (EAEC), *Campylobacter jejuni* (particularly in Southeast Asia), are still identified most frequently in patients abroad with AGE/TD, while *Shigella* spp. and *Salmonella* spp. have lately become less frequent in on-site assessment, but may persist in returning travelers [2, 7, 19, 20, 22–26].

Parasitic infections, such as *Giardia lamblia*, are generally less frequent but important causes of persistent gastrointestinal symptoms and may contribute a relatively larger proportion of post-travel diarrhea cases [8, 27]. Awareness of viral causes of travel-acquired AGE has increased along with improved and more readily accessible diagnostic methods. However, under-ascertainment remains a concern because of limited post-travel healthcare-seeking behavior due to factors such as differences in the clinical course of infection (i.e., relatively milder severity and chronicity of infection) and limited viral testing in both clinical practice and research.

Noroviruses are a leading cause of AGE globally across all age groups and have, further, been identified as an important cause of non-bacterial TD and AGE among travelers [21, 28–35]. While norovirus incidence rates among travelers are rarely reported, prevalence estimates among individuals with TD and/or AGE range from 3 to 65% depending upon factors such as diagnostic methods, population, and viral activity level, and co-infections are commonly reported [7, 21, 28, 31, 36, 37]. Of the seven recognized norovirus genogroups, genogroup I (GI) and II (GII), cause the majority of human illness with genotype GII.4 responsible for most outbreaks in recent years [38–40]. It is important to recognize that norovirus causes vomiting in a substantial proportion of cases in the absence of diarrhea, thus the term AGE is most relevant for describing clinical symptoms related to this infection. Further, vomiting may facilitate transmission in contained settings, such as cruise ships, where norovirus has been identified as the responsible pathogen in approximately 97% of AGE outbreaks reported to the US-based Vessel Sanitation Program [41, 42]. Norovirus transmission in such situations can be difficult to control due to its low infective dose, environmental hardiness, and multiple routes of transmission (person-to-person as well as through contaminated food, water, and/or fomites) [43, 44]. Sporadic/endemic norovirus-related illness is common, as shown through cohort-based community studies, as is viral detection in the environment [45–56]. Viral evolution and possible genotype-specific waning of immunity following natural infection place adult travelers, and non-travelers alike, at risk of acquiring novel norovirus infections and subsequent illness (withstanding the possibility for a certain level of innate protection against specific norovirus genotypes due to genetic determinants of histo-blood group antigens (HBGAs) on the gut epithelial surfaces) [51, 57, 58]. Further, travelers may in fact be important sources of global norovirus circulation and the introduction of new outbreak strains to new geographic locations [59–61].

Increased risk of post-infectious functional gastrointestinal disorders (PI-FGD), particularly irritable bowel syndrome (IBS), and other potential PI sequelae such as rarely Guillain-Barré Syndrome, have been reported among individuals who experienced TD or infectious diarrhea [62–71]. While excess PI-FGD disorders occur following both bacterial and viral enteric infections, including norovirus, there remains some controversy regarding the strength of the association and characterization of important host, pathogen, and environmental risk factors [72–77].

### Knowledge gaps

This study seeks to address several key gaps in knowledge related to the role of norovirus as a cause of travel-acquired AGE. While numerous studies have documented the important role of AGE among international travelers, a full account of norovirus’ contribution to rates of travel-acquired infections, illness, healthcare utilization, daily function, costs, and PI sequelae are needed to define targeted interventions. Prior studies that utilized a case definition for AGE that did not capture isolated vomiting (without diarrhea) are not ideally suited to fully capture norovirus [42]. Many studies reporting the potential contribution of norovirus, have focused on ill travelers who seek post-travel healthcare thereby missing cases with or without medical care while abroad and may suffer from under ascertainment of viral causes of illness due to lack of testing and/or possible clearance relative to non-viral enteric pathogens [1, 6, 13, 21, 78–80]. The existing studies do not provide sufficient data to calculate incidence rates, describe norovirus genotype distribution, the presence of co-infections, discern travel versus non-travel-acquired infections/illnesses, nor describe traveler characteristics associated with norovirus AGE. There is also a lack of information on the impact of norovirus AGE on travel plans due to illness. Location-based prospective studies of gastrointestinal infections identified and diagnostically-confirmed at pre-defined travel destinations have provided important information on specific populations and/or destinations, but may be limited in generalizability, viral detection methods, or ascertainment of pre-travel health status [2, 20, 81–84].

Our aim is to identify the burden of AGE, particularly those cases caused by norovirus, among travelers leaving from North America and Europe to areas at moderate to high risk of traveler’s diarrhea by utilizing a prospective design that enables attribution of infection to the travel period. Data generated by this study will be useful for guiding future development and testing of prophylactic and/or therapeutic agents targeting travel-acquired AGE with a particular focus on prevention of norovirus AGE.

## Methods / design

The norovirus travel study is a prospective multi-site cohort study of AGE and norovirus risk among adult international travelers from the US and Europe to areas of moderate to high risk of traveler’s diarrhea. The overall aim of the study is to estimate the burden of AGE caused by norovirus acquired while traveling internationally.

We utilized broad eligibility criteria according to study population characteristics such as age and health status, as well as travel type and destination. We chose to limit eligible trip length to 3–15 days to allow travelers to provide post-travel stool samples within 14 days of AGE symptom onset, when applicable, which was considered reasonable for the detection of norovirus based on available viral shedding data [85–88]. This study design enabled centralized diagnostic testing irrespective of travel destination thereby increasing the diversity of travel type and destination. This prospective study of AGE among international travelers is the first to assess laboratory-confirmed norovirus, based on pre- and post-travel stool samples, among both symptomatic and asymptomatic travelers and to assess a wide set of burden of disease measures among travelers from Europe and North America.

The primary study objective is to:

- estimate the incidence of AGE due to travel-acquired norovirus.

Specific secondary research objectives are to:

- estimate the incidence of AGE due to norovirus according to risk factors related to host (including age, gender, underlying health conditions) and environment (including travel origin, destination, and mode of travel)
- estimate the incidence of medically-attended (outpatient and inpatient) AGE due to norovirus
- estimate the proportion of AGE due to norovirus as single and/or co-infections
- estimate the impact of AGE due to norovirus on daily functioning and travel plans
- describe the distribution of norovirus genotypes associated with travel, overall as well as by region of origin and travel destination
- describe travel behaviors (including type of travel and eating/drinking-related behavior) associated with AGE due to norovirus, and to
- describe the clinical course (severity, duration, and sequelae/new onset conditions) of AGE due to norovirus, taking into consideration cofactors such as age, underlying health conditions, and medication use.

Exploratory research objectives are to:

- estimate the incidence of asymptomatic norovirus infections
- estimate the direct and indirect costs associated with AGE due to norovirus
- describe additional and secondary AGE cases among traveling and non-traveling companions and household members, and to
- assess the impact of delayed stool collection relative to AGE symptom onset on the detection of norovirus and other enteric pathogens.

This study was approved by the respective ethical review board at each study site. Electronic case report forms will be used within a centralized electronic data capture system that provides an audit trail for data entry. Remote and on-site monitoring of study implementation is ongoing throughout the study.

### Study population

**Fig. 1:**
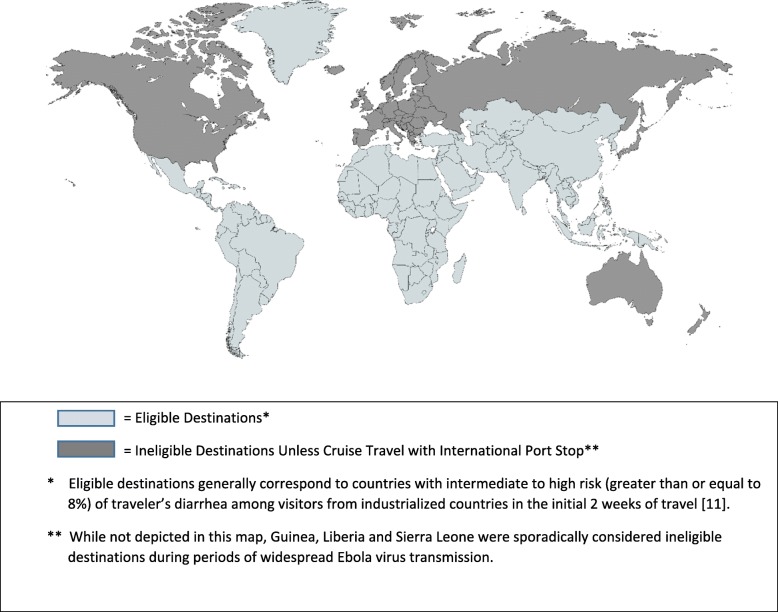
Norovirus travel study: global map depicting eligible and ineligible travel destinations

The study population consists of individuals ≥18 years who are residents of Germany, Switzerland or the United States, are fluent in German and/or English, and travel internationally for 3–15 days beginning within 120 days of enrollment. Eligible travel includes destinations other than Europe (defined according to the 2013 United Nations Statistics Division Standard Country and Area Codes Classification [89]), the United States of America, Canada, Japan, Australia or New Zealand or cruise ship travel that includes an international port stop in any country other than country of origin (Fig. 1). All study participants provided signed informed consent and were received nominal compensation for completing key study activities. Study participants were excluded if their international travel destination was to an area of high Ebola transmission at the time of enrollment (defined by the Centers for Disease Control and Prevention (CDC) as an area with high Ebola transmission and concomitant “Warning Level 3, Avoid Nonessential Travel” designation [90]).

Between March 2015 and April 2017, 1386 study participants were enrolled from travel clinics and community advertising located at the following five sites: Division of Infectious Diseases and Tropical Medicine University of Munich, Munich Germany; Travel Health Centre at the Epidemiology, Biostatistics and Prevention Institute, University of Zurich, Zurich Switzerland; Swiss Tropical and Public Health Institute, Basel Switzerland; Rollins School of Public Health, Emory University, Atlanta, Georgia, United States of America; and Kelsey Research Foundation/School of Public Health University of Texas, Houston, Texas, United States of America. Study-targeted travel spanned approximately 27 months with travel destinations to nearly every region of the world.

### Data collection and follow-up of study participants

Table 1 depicts key data collection events throughout the study. Each participant was expected to contribute between 1 and 11 month(s) of person-time. All participants were expected to provide baseline information, daily travel diaries, and diaries at post-travel days 2, 7, and 14, as well as pre-travel stool samples within 7 days of travel start. Both paper and electronic diaries were utilized in this study. A subset of asymptomatic travelers, as well as all travelers who experienced AGE with an onset between day 2 of travel and day 2 post-travel, were requested to provide a post-travel stool sample and were contacted at 3 and 6-months after their return to provide health information post-travel.

**Table 1:** Norovirus travel study: data collection event summary

Content	Data Collection Event									
	Pre-Travel			Peri-Travel	Post-Travel					
	Recruitment	Baseline	Clinical Sample^a^	Daily Diary	Diary Day 2	Diary Day 7	Diary Day 14	Clinical Sample^b,^^c^	3 Month Follow-Up^c^	6 Month Follow-Up^c^
Eligibility	●									
Demographics	●									
Travel										
Plans/Itinerary	●	●		●	●					
Type/Reason for Travel		●								
Behavior/Activities				●	●					
Past 3 Months		●							●	●
Health Information										
Blood Type		●								
Pre-Existing Condition		●								
Regular Bowel Patterns		●							●	●
Non-Specific Symptoms		●		●	●	●	●		●	●
Illness in Week Prior to Travel^*^				●						
Medication/Vaccination										
Medications		●		●	●	●	●		●	●
Vaccinations		●		●	●	●	●			
Acute Gastroenteritis		●		●	●	●	●		●	●
Week Prior to Travel^*^				●						
Medical Care				●	●	●	●		●	●
Direct and Indirect Costs						●	●		●	●
Impact on Daily Function				●	●	●			●	●
Impact on Travel Plans / Planned Activities				●	●	●				
Travel Companion and Household Illness							●			
New Onset Conditions / Potential Post-Infectious Sequelae									●	●
Medical Care									●	●
Direct and Indirect Costs									●	●
Impact on Daily Function									●	●
Self-Collected Stool			●					●		

^a^Pre-travel clinical sample provided within 7 days of travel start

^b^Post-travel clinical sample provided within 14 days of symptom onset if traveler experienced acute gastroenteritis between day 2 of travel and day 2 post-travel or within 14 days of travel return if traveler was selected at study enrollment to provide a post-travel stool sample regardless of the absence of acute gastroenteritis symptoms while traveling

^c^Data collection event for a subset of the study participants including those with acute gastroenteritis between day 2 of travel and day 2 post-travel or 25% of study participants selected at enrollment to provide post-travel stool samples and extended 3 and 6-month follow-up

Study participants were considered lost to follow-up if the participant did not provide the expected study visit/interview and/or stool sample and the site staff were unable to reach the participant after three attempts. In this situation, person-time was censored from the date of the last data point provided.

### Stool sample collection and diagnostic methods

Self-collected whole stool samples were requested from all participants within 7 days prior to travel initiation (pre-travel stool samples). This sample was collected in a single vial. Post-travel stool samples were requested from all study participants who experienced any vomiting and/or diarrhea between day 2 of travel and day 2 post-travel. These symptomatic individuals provided a self-collected whole stool sample after returning from their trip and within 14 days of symptom onset. Further, at the time of enrollment, each site assigned every fourth participant to the ‘asymptomatic’ subset, and these subjects were asked to provide a self-collected whole stool sample within 14 days of travel return even in the absence of vomiting and/or diarrhea. This sampling algorithm assumed that 20% of such participants would, in fact, experience such symptoms while traveling and would be expected to provide a post-travel stool sample within 14 days of symptom onset. All travelers who provided a post-travel stool sample were asked to provide a single sample collected in two separate vials.

All post-travel stool samples (vial 1) were tested via real-time quantitative reverse transcription-polymerase chain reaction (RT-qPCR) at a central laboratory for genogroup I and genogroup II noroviruses. If the post-travel stool sample was RT-qPCR norovirus positive, the paired pre-travel stool sample from the study subject was tested by the same diagnostic method. A systematic random sample (approximately 50%) of post-travel stool samples (vial 2) obtained from travelers who experienced travel-acquired AGE was tested via Luminex xTAG® Gastrointestinal Pathogen Panel (GPP) for multiple enteric pathogens. The RT-qPCR norovirus results will be used for the key study objectives, and samples with norovirus positive results (using a cycle threshold value of < 40) were tested via conventional RT-PCR for sequence analysis of the amplicon targeting open reading frame 1 and 2 (regions C and D). Comparisons between RT-qPCR and Luminex xTAG® GPP norovirus results will also be possible.

**Fig. 2:**
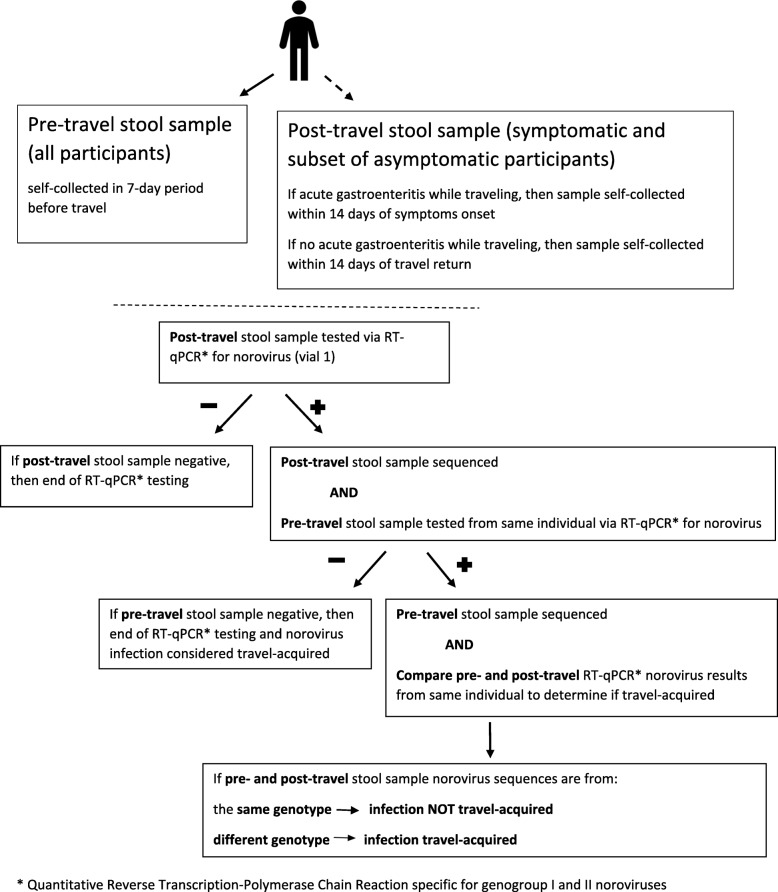
Stool sample collection and testing strategy and algorithm for considering norovirus infection to be travel-acquired

**Fig. 3:**
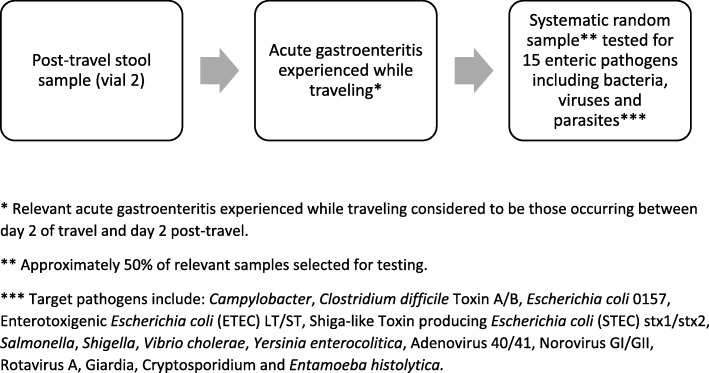
Diagnostic testing strategy for enteric pathogens using the Luminex xTAG® Gastrointestinal Pathogen Panel

All stool samples were self-collected in vials without cryopreservative and stored in refrigerated conditions until delivery to a study site (within 24 h), at which time they were frozen at − 70 degrees Celsius or colder. Pre-travel stool samples and one vial of the post-travel stool samples were shipped frozen to a central laboratory for RT-qPCR norovirus testing. Vial 2 of the post-travel stool sample were available for possible enteric pathogen testing via the Luminex xTAG® GPP, a multiplex PCR test that can identify: *Campylobacter*, *Clostridium difficile* Toxin A/B, *Escherichia coli* 0157, Enterotoxigenic *Escherichia coli* (ETEC) LT/ST, Shiga-like Toxin producing *Escherichia coli* (STEC) stx1/stx2, *Salmonella*, *Shigella*, *Vibrio cholerae*, *Yersinia enterocolitica*, Adenovirus 40/41, Norovirus GI/GII, Rotavirus A, Giardia, Cryptosporidium and *Entamoeba histolytica*. The diagnostic testing strategy for norovirus and other enteric pathogens is depicted in Figs. 2 and 3.

### Case definitions and data source for key study objectives

AGE was defined as any vomiting, or three or more loose or watery stools, or two or more loose or watery stools plus the presence of at least one additional symptom (such as fever, abdominal cramps, urgency, nausea) within 24 h.

AGE was considered as travel-acquired and due to norovirus if a study participant experienced AGE (as defined above) and the AGE symptoms began between the second day of travel and the second day after travel return, and the pre-travel stool specimen was norovirus negative and the post-travel stool specimen was norovirus positive, or if both stool specimens were norovirus positive but the genotypes differ.

Medically-attended AGE due to norovirus (as described above) that was acquired while traveling was identified via self-reported healthcare contacts (see Table 1 for collection time-points) categorized as: telephone consults, pharmacy consults, emergency/urgent care, non-urgent outpatient consults, home health care, traditional healer and hospitalizations. Abstraction of personal copies of post-travel hospitalization records provided by the study participants were utilized when available.

The impact of travel-acquired AGE due to norovirus (as described above) on daily function and travel plans were similarly, identified via self-report (see Table 1 for collection time-points). Impact on daily function, if present, was defined according to level of incapacitation (restriction to lodging, partially or completely bed-ridden), as well as, perceived impact on functioning classified as mild, moderate or severe. The impact of travel-acquired AGE on travel plans was classified according to change and/or cancellation of plans and the duration of such interruption of plans.

### Sample size and analysis plan

Sample size calculations were based on the normal approximation of the binomial proportion confidence interval to estimate proportions with 95% confidence and 1% precision. The proportion of primary interest is the proportion of travelers with AGE due to norovirus. A summary of sample size calculations and assumptions are presented in Table 2. Our goal is to accurately estimate the proportion of international travelers from US and European-based study sites who acquire symptomatic norovirus based on assumptions that 10 to 40% of such travelers will experience the onset of AGE symptoms between day 2 of travel and day 2 post-travel and that 2.5 to 10% of such cases will be due to norovirus.

**Table 2:** Sample size calculations for estimating the proportion of travelers with acute gastroenteritis due to norovirus

Probability of Norovirus given Acute Gastroenteritis	Probability of Acute Gastroenteritis			
	10%	20%	30%	40%
2.5%	250	499	744	991
5%	499	991	1477	1958
10%	991	1958	2904	3830

Note: Assumptions: 1) Confidence Interval = 95%, 2) Precision = 1%, 3) Separate Regional Estimates Based on Study Site (European and United States), and 4) 30% Over-Enrollment to Account for Incomplete Follow-Up

Key study objectives will be analyzed according to a formal statistical analysis plan. Data will be initially summarized according to person-time, compliance with expected study milestones (missing data, loss-to-follow-up and adherence with expected timing of data provided) and descriptive statistics related to the study cohort, their travel and health status across follow-up time, as well as summary data pertaining to clinical sample compliance and results. The impact of incomplete compliance with the study protocol will be assessed through comparison of full and partial analytical datasets. Incident infections and AGE will take into consideration prevalent infections and symptoms. Norovirus AGE incidence calculations will be restricted to those individuals who have provided both a pre- and post-travel stool sample. Stratified analyses and multivariable regression modeling may be used to calculate incidence rates that identify and account for potential confounding and effect modification by key study variables. Misclassification of key variables will be assessed through sensitivity analyses based on alternate data sources and assumptions as appropriate. Interpretation of statistical measures, including confidence intervals, will be tempered with appropriate judgment and acknowledgments of potential sources of error and limitations of the analysis.

## Discussion

We present a unique study design for a broad assessment of the burden of AGE due to norovirus acquired during international travel. We have utilized a prospective cohort study design that identifies study participants prior to travel and is not limited to medically-attended ill returning travelers. This will allow incidence data to be captured for both medically-attended and non-medically-attended AGE events as well as description of the severity and course of illness among study participants. The strengths of this study are briefly described below as they relate to the study objectives described in the methods/design section of this manuscript.

### Incidence of travel-acquired AGE due to norovirus: Overall, according to host/environmental factors, and medically-attended

The design of this study will allow prevalent and incident norovirus infections to be differentiated due to the collection of pre- and post-travel whole stool samples for norovirus testing via RT-qPCR. The capture of AGE symptoms before, during, and after travel will be used to establish the timing of AGE cases. We used a broad case definition for AGE inclusive of vomiting alone and/or TD. This is expected to capture a substantial proportion of norovirus cases that cause vomiting in the absence of diarrhea [42]. We will be able to assess the influence of age, gender and underlying health on incident norovirus AGE, as well as, the influence of travel origin/destination and mode given the collection of demographic and health information prior to travel. Further, we will calculate the incidence, and type of medically-attended norovirus AGE (phone, pharmacy consult, traditional healer, outpatient, hospitalization) based on health care utilization data collected on travel/post-travel diary forms.

### Norovirus detection, genotype distribution, and co-infections with other pathogens among symptomatic and asymptomatic travelers

The use of self-reported diaries to capture illness symptoms along with the collection, and RT-qPCR testing, of paired pre- and post-travel stool samples will allow us to estimate the burden of travel-acquired AGE and the proportion of such cases that are due to norovirus. Given the potential for asymptomatic norovirus infections, every 4th subject enrolled in the study has been asked to provide a post-travel stool sample (in addition to the pre-travel sample to be provided by all travelers) so that we can estimate norovirus incidence and characterize viral outcomes in these travelers [87, 91, 92].

We have designed this study to utilize a standard, centralized RT-qPCR-based diagnostic testing algorithm to identify and genotype norovirus cases. Norovirus genotype distribution will be described overall, as well as by travel origin and destination, which will add valuable information for travel-acquired norovirus given the anticipated wide range of travel destinations. In addition to centralized norovirus testing, we will assess the role of other common enteric pathogens in travel-acquired AGE via regional testing of a systematic random sample of post-travel stool samples from travelers with AGE via Luminex xTAG® GPP. These results, in conjunction with the RT-qPCR results, will provide an estimate of the proportion of AGE cases that have norovirus identified with and without additional enteric pathogens present.

### Travel behaviors associated with norovirus AGE and impact of norovirus AGE on daily functioning and travel plans

In addition to data collected on health status while traveling, study participants will provide information related to the type of travel, daily travel behavior, and the potential impact of AGE on daily functioning and adherence to travel plans. Given the collection of this information, we will be able to assess the role of traditional AGE/TD risk factors on norovirus AGE and the impact of such illness which will provide information to characterize the impact of norovirus AGE among international travelers and help guide prevention efforts.

### Clinical course of norovirus AGE

Health-related data collected on travel diaries and post-travel diaries/interviews will allow us to assess self-reported illness severity, duration, and possible post-infectious sequelae/new onset conditions up to 6-months post-travel (data collected during the first 2 weeks post-travel, as well as, at 3 and 6-months post-travel). We will be able to compare potential sequelae for individuals with, and without, travel-acquired norovirus AGE due to our collection of comparable data for a subset of asymptomatic individuals. This information on laboratory-confirmed norovirus AGE is expected to fill a current gap in knowledge among the international travel population.

### AGE cases among traveling and non-traveling companions and household members

The inclusion of post-travel data from study participants related to AGE symptoms among their travel companions and non-traveling household members will provide information on the potential for secondary AGE cases and sustained transmission of norovirus due to imported cases. This information, while not laboratory-confirmed, is expected to broaden our current knowledge related to the full impact of travel-acquired norovirus AGE.

Despite the unique strengths of our study design, there are some potential limitations and considerations with respect to the interpretation of the ensuing results which are summarized below. The first concerns choice of study population and travel characteristics. The study population is a broad composition of adults, without health-based restrictions, attending travel and non-travel health clinics, as well as individuals recruited through community and college-based advertising. There were no restrictions on type of travel, which is expected to result in a range of potential exposures. The broad inclusion criteria used in this study will provide data that we anticipate will be generalizable to a broad group of international travelers from the US and Europe.

Our choice of eligible travel destinations has both advantages and limitations. Eligible travel destinations, e.g., countries in regions generally considered to have intermediate/high risk of TD among visitors, may coincide with travel destinations targeted by other travel vaccines and/or preventive travel-related health services due to heightened disease risk which may increase the generalizability of our study population to international travelers who visit travel clinics. It is, however, understood that many intestinal infections, such as norovirus, are not limited to lower-resource settings and can have high transmission in any setting given the right conditions. Further, given the restriction of travel destination to areas with higher risk of TD, it may also be expected that within this study population there may be a high proportion of coinfections amongst those with and without AGE. However, we may have reduced sensitivity to detect bacterial AGE cases due to the potential delay in collection and testing of stool samples and the absence of culture-based detection methods. Our study will include international travel with stops in both eligible and non-eligible destinations. While the impact of inclusion of such travel can be accounted for in the analyses through stratification, pooled analyses may dilute risk estimates if there are differences between high- and low-resource travel destinations. The inclusion of cruise ship travel with international port stops regardless of region may also dilute study findings in a pooled analysis if AGE risk is low in cruise destinations that include North America and Europe relative to other eligible travel destinations but can be assessed in stratified analyses. The choice of travel duration between 3 and 15 days was considered a balance between our ability to capture a reasonable proportion of international travelers and the likelihood of being exposed to and (developing symptoms of) norovirus during a given trip, the ability to collect stool samples during the time period when viral shedding occurs, and to reduce recall bias [85–87, 93–98]. We recognize that many international trips are for periods greater than 15 days and that international trip length may differ according to regional norms and/or travel situation (e.g., expatriate, humanitarian, student, and military assignments), but incidence of AGE is considered to be highest during the first 2 weeks of travel [11]. The use of improved detection methods on stored/stable stool samples, such as molecular testing of stool collected on hemoccult cards, may prove useful for future observational and/or clinical studies [99].

We define AGE onset to be travel-acquired when symptom onset is reported to begin between day 2 of travel until day 2 following travel return. This time window maximizes the capture of norovirus AGE which has an average incubation period of approximately 33 h [100]. It is possible, however, that this time window choice may include some domestically-acquired norovirus AGE cases or AGE cases caused by other enteric pathogens with short incubation periods. Pre- and post-travel stool sample collection timing was also selected to optimize the attribution of norovirus-related AGE to the travel period. The pre-travel sample, collected within 7 days of travel start, was chosen to identify prevalent infections. The choice of timing was selected as a balance between realistic expectations of study participants during the busy pre-travel period and the ability to minimize the attribution of illness occurring early in a trip to infections that were acquired before the travel began. The post-travel sample timing was selected to be within 14 days of AGE symptom onset. The asymptomatic subset asked to provide a post-travel stool sample are asked to provide a post-travel sample within 14 days of travel return. Ideally, the stool sample would be collected as soon as possible after symptom onset to ensure detection of the causal pathogen if present. However, we chose to ask study participants to provide their second stool sample after travel return given our priority to include a broad set of travelers and travel destinations and the difficulties (validation and cost) related to the collection and transport of stable stool samples from various destinations or an alternate option of establishing standardized testing facilities in all possible destinations. The collection of post-travel stool samples after returning home was made based on a review of the literature which suggests that norovirus is excreted and detectable in stool for both symptomatic and asymptomatic infections for several weeks after infection [85, 86]. While we accounted for 30% non-compliance for provision of a post-travel stool sample in our sample size estimates (Table 2), the possibility exists that travelers with and without AGE may differentially comply with this study requirement. This can be assessed in our analyses, however, given the inclusion of a subset of travelers who were asked to provide a post-travel stool sample regardless of the presence of AGE symptoms while traveling. We chose to utilize RT-qPCR as the gold standard for norovirus detection while also testing a systematic random sample of post-travel symptomatic stool specimens via Luminex xTAG® GPP to identify possible co-infections. The multiplex test has the ability to detect 15 common pathogens, has been found to have high sensitivity and specificity, and is expected to provide valuable information on pathogens other than norovirus amongst the study participants [101, 102]. There are limitations, however, as the panel does not include all pathogens that may be encountered/expected amongst travelers. In addition, it is expected that the test performance may have pathogen-specific variability associated with variability in time lag between symptom onset and stool sample collection.

An additional feature of this study that warrants discussion is the use of active data collection and the breadth of data captured. All participants were asked to provide baseline data, daily travel diaries, and 3 separate diary entries post-travel day 2, 7 and 14 for information related to travel behavior and health status. Compliance with diary provision will be assessed according to travel and host characteristics. Post-travel data collection at 3 and 6 months will provide valuable information on new onset health conditions, such as PI-FGD, as well as, course of illness for those who experienced AGE and may have extended health effects. Assessment of prolonged symptoms and new onset health conditions for a period up to 6 months post-travel aligns well with the time period assessed in prior studies, practical considerations of tracking travelers over an extended period, and appropriate time period to assess the onset of potential complications given the biologic plausibility of many PI-FGD [62–64, 67, 74, 103, 104]. We acknowledge that this study protocol could have included the capture of an additional clinical sample (e.g., saliva) to assess the influence of HBGAs and secretor status on norovirus incidence, however due to practical considerations we did not include this analysis in the current study.

Active data collection before, during, and after travel, while intensive, will provide critical information on health status regardless of healthcare-seeking behavior, but this requires a high level of compliance among the travelers. Nominal compensation was provided to participants to counter the amount of time and effort needed for the expected study activities, but there remains the potential for sub-optimal follow-up or differential compliance.

## Conclusion

AGE remains the most common illness of travelers while abroad or returning from low-income destinations. The contribution of norovirus, and specific norovirus genotypes, to incident travel-acquired AGE among a diverse study population will be investigated in this prospective study among adults traveling internationally for 15 days or less. It will further quantify the impact of AGE, both overall and due to norovirus, on daily function and travel plans. The collection of data on asymptomatic travelers and the use of PCR-based diagnostic methods to identify incident infections will further our understanding of the causal role of norovirus in travel-acquired infections. This study will assess post-travel health conditions for travelers with and without AGE. Knowledge gained will be useful for the development, and potential future testing of, preventive and therapeutic agents targeting travel-acquired AGE, those cases caused by norovirus, as well as potential travel advice.

## Abbreviations

AGE: Acute gastroenteritis
CDC: Centers for Disease Control and Prevention
EAEC: Enteroaggregative *Escherichia coli*
ETEC: Enterotoxigenic *Escherichia coli*
GPP: Gastrointestinal pathogen panel
HBGAs: Histo-blood group antigens
PI: Post-infectious
PI-FGD: Post-infectious functional gastrointestinal disorders
RT-qPCR: Real-time quantitative reverse transcription-polymerase chain reaction
STEC: Shiga-like toxin producing *Escherichia coli*
TD: Traveler’s diarrhea
US: United States
